# Vaccination with bispecific antibody armed T cells (BATC) in metastatic breast cancer patients and transfer of anti-breast cancer immunity in primed T cells after stem cell transplant: a proof of principle study

**DOI:** 10.1186/2051-1426-3-S2-P56

**Published:** 2015-11-04

**Authors:** Archana Thakur, Lawrence G Lum, Vidya Kondadasule, Ritesh Rathore, Zaid Al-Kadhimi, Elyse Tomaszewski, Dana Schalk, Joseph Uberti, Voravit Ratanatharathorn

**Affiliations:** 1 Karmanos Cancer Institute, Wayne State University, Detroit, MI, USA; 2 Roger Williams Hospital, Providence, RI, USA; 3 Emory University School of Medicine, Atlanta, GA, USA

## Background

Despite improvements in treatment options, metastatic breast cancer (MBC) remains an incurable disease. In our recent Phase I immunotherapy (IT) trial in 23 women with MBC, 8 infusions of activated T cells (ATC) armed with anti-CD3 x anti-HER2 bispecific antibody (HER2Bi) given in combination with interleukin-2 (IL-2) and granulocyte-macrophage colony stimulating factor (GM-CSF) induced specific anti-breast cancer (BrCa) cytotoxicity and increased IL-12 and Th_1_ cytokines in the serum^1^. This study investigated whether specific cellular and humoral anti-BrCa immunity is induced by infusions of HER2Bi bispecific antibody armed T cells (BATs) could be transferred after high dose chemotherapy (HDC) and stem cell transplant (SCT).

## Methods

T cell were activated with OKT3 and expanded in IL-2. ATC were harvested, armed with HER2Bi, and cryopreserved in 8 doses for twice weekly infusions for 4 weeks along with IL-2 and GM-CSF. Seven to 14 days after the last infusion of BATs, the patient was leukapheresed to obtain immune T cells. Immune ATC were harvested and cryopreserved for multiple infusions after the HDC and SCT. Cellular and humoral immune responses were monitored up to 24 months.

## Results

Six of 8 MBC patients enrolled in the protocol, completed the protocol and were evaluable for transfer of cellular and humoral immunity. No dose-limiting events for the infusions, delays in engraftment, and life-threatening infections were observed. Five of 6 evaluable patients exhibited increased anti-BrCa cytotoxicity and IFN-γ Elispots after vaccination with BATs and up to 12 months post SCT. Serum and culture supernatantsfrom *in vitro* antibody synthesis assay showed gradual increases in anti-SK-BR-3 IgG levels after SCT. Serum cytokine profile showed increases in IL-12 and Th_1_ cytokines. One of 6 evaluable patients who rapidly progressed showed poor immune responses (CTL and IFN-γ Elispots), had high serum levels of Th_2_ cytokines and no evidence of transfer of immunity. Flow cytometry analysis of Vβ repertoirepattern in PBMC collected post IT and post SCT indicate transfer of the major Vβ clones post SCT.

## Conclusions

This pilot study suggests that optimal adoptive transfer of cellular and humoral immunity induced by BAT infusions using *ex vivo* expanded immune anti-breast cancer T cells after SCT accelerates not only immune reconstitution but, more importantly, enhances reconstruction of anti-tumor cellular and humoral immunity after HDC and SCT to achieve maximal tumor reduction and regulatory T cell ablation.
